# VEGFR Blockade Reduces *Mycobacterium tuberculosis*-Induced Lung Pathology in Immunocompromised Mice

**DOI:** 10.3390/cells15070573

**Published:** 2026-03-24

**Authors:** Melinda Herbath, Jeffrey Harding, Thanthrige Thiunuwan Priyathilaka, Collin James Laaker, Athena Kafkas, Zsuzsanna Fabry, Matyas Sandor

**Affiliations:** 1 Department of Pathology and Laboratory Medicine, School of Medicine and Public Health, University of Wisconsin-Madison, 1111 Highland Avenue, Madison, WI 53705, USA; jharding@lunenfeld.ca (J.H.); thanthrige@wisc.edu (T.T.P.);; 2 Neuroscience Training Program, University of Wisconsin-Madison, 1111 Highland Avenue, Madison, WI 53705, USA; claaker@wisc.edu

**Keywords:** *Mycobacterium tuberculosis*, immunocompromised, neutrophil granulocytes, VEGFR1, SU5416

## Abstract

**Highlights:**

**What are the main findings?**

- VEGFR1/2 blockade using SU5416 (semaxanib) reduces lung pathology in immunocompromised (RAG1KO) mice infected with *Mycobacterium tuberculosis* (Mtb), as evidenced by decreased inflamed areas and lower numbers of monocytes and NK cells, without affecting bacterial burden.
- An elevated ratio and increased absolute number of neutrophils were observed in Mtb-infected lungs following SU5416 treatment. This was seen in both immunocompromised and immunocompetent mice. At this phase of the infection, most neutrophils remained within the lung vasculature; they did not infiltrate tissue and did not exacerbate lung pathology. This mechanism appears to be VEGFR1-independent.

**What are the implications of the main findings?**

- The data supports that VEGFR blockade with SU5416 shows promise as a host-directed therapy for TB, particularly in immunocompromised individuals, by reducing lung pathology while preserving protective immunity.

**Abstract:**

*Mycobacterium tuberculosis* (Mtb) remains a significant public health threat, responsible for 1.6 million deaths in 2021. The development of new treatments is particularly urgent for immunocompromised individuals, including those with Mtb/HIV coinfection, who experience severe disease outcomes. Previous studies demonstrated that blockade of VEGFR1, a receptor expressed on monocytes that mediates their recruitment to infection sites, limits Mtb-induced pathology in immunocompetent mice of both Mtb-resistant (C57BL/6J) and Mtb-susceptible (B6.C3H-sst1) strains. The present study extends these findings by evaluating the VEGFR1/2 blockade strategy in immunocompromised hosts. Treatment with the VEGFR1/2 blocker SU5416 (semaxanib) reduced monocyte infiltration into the lungs of Mtb-infected immunocompromised RAG1KO mice without affecting bacterial protection. Reduced monocyte recruitment improved lung pathology. VEGFR1/2 blockade also decreased the number of NK cells in the lungs of RAG1KO mice. Notably, an elevated ratio and increased absolute number of neutrophil granulocytes were observed in the Mtb-infected lungs of both immunocompetent and immunocompromised mice following SU5416 administration. However, this increase in neutrophils did not exacerbate lung pathology, as most recruited granulocytes remained within the lung vasculature. The beneficial effect of VEGFR1/2 blockade in RAG1KO animals suggests that further investigation of VEGFR blockers, such as SU5416, as adjunctive therapy to anti-tuberculosis drug regimens for immunocompromised populations with tuberculosis is warranted.

## 1. Introduction

Despite global efforts, tuberculosis (TB) remains one of the deadliest diseases. In 2022, it was the world’s second leading cause of death from a single infectious agent, only surpassed by COVID-19. Globally in 2022, TB caused an estimated 1.30 million deaths (95% UI: 1.18–1.43 million), almost twice as many deaths as HIV/AIDS [1]. Due to their weakened immune system, people living with HIV are up to 20 times more likely to fall ill with TB. Among people with latent TB infection, HIV infection is the strongest known risk factor for progressing to TB disease [2]. Additionally, treatments such as steroids, chemotherapy, or biologics that impair the host’s adaptive immune mechanisms can also increase the risk of developing active TB disease [3]. Due to their lack of adequate CD4+ T cell response, HIV-TB patients are at a unique immunological disadvantage and depend on the efficiency of innate immune cell populations, including monocytes, macrophages, neutrophils, and NK cells, to control Mtb infection. It has been shown that *Mycobacterium tuberculosis* (Mtb)/HIV coinfection results in an excessive pro-inflammatory response, leading to poorly formed granulomas, more severe lung pathology, and increased mycobacterial burden and dissemination in human patients [4,5] and in humanized mice [6].

Animal studies using RAG-deficient mice, which lack mature T- and B-lymphocytes, have been extensively used to evaluate the protective role of innate immune response in TB infection. It has been shown that RAG mice are unable to control mycobacterial infection and display high bacterial burden, but adoptive transfer of CD4+ T cells into deficient hosts confers protection [7] and promotes granuloma formation [8], as CD4+ T cells are central mediators of granuloma formation and maintenance [9,10,11]. It is long known that IFN-gamma production by CD4+ T cells is indispensable for controlling Mtb infection [11,12]. IFN-gamma-producing NK cells increased in the lungs of cynomolgus macaques during Mtb infection, indicating that NK cells may contribute to host responses in TB [13]. Although immunodeficient animal models have been widely used to study Mtb host responses, research on adjunct host-directed therapies in immunocompromised mice remains limited.

Vascular endothelial growth factor A (VEGFA) blockade as a host-directed therapy (HDT) was proposed previously [14]. It was shown that partial VEGFA blockade normalizes vasculature around granulomas and improves drug delivery and immune cell access and function [15]. However, it was pointed out that anti-VEGF treatment is effective alone but is used as a host-directed adjunct therapy in synergy with antibiotics. We have previously shown that (VEGFA) is an important contributor to Mtb-induced lung damage by promoting the excessive recruitment of pro-inflammatory monocytes into the infected lungs. VEGFA, a growth factor primarily known for its roles in angiogenesis, binds to KDR/VEGFR2 receptors [16,17] on endothelial cells and induces endothelial cell proliferation, promotes cell migration, inhibits apoptosis, and promotes permeabilization of blood vessels [18,19]. Additionally, VEGFA functions as a chemoattractant for monocytes and promotes macrophage migration through FLT1/VEGFR1 that is expressed by this cell population [20]. Our previous results show that granuloma macrophages are the main source of VEGFA and that myeloid-cell-targeted blockade of VEGFA production or blocking VEGFR1 on monocytes can reduce Mtb-induced pathology in immunocompetent mice [21].

Given the significant risk that tuberculosis poses to immunocompromised populations, this study investigated whether the beneficial effects of VEGFR blocker treatment, previously described in immunocompetent mice [21], are also observed in immunocompromised RAG1KO animals. The results demonstrate that treatment with the VEGFR1/2 inhibitor SU5416 reduces lung inflammation in RAG1KO mice lacking an adaptive immune system without compromising bacterial protection. VEGFR1/2 blockade decreases monocyte and NK cell accumulation while increasing both the relative and total numbers of neutrophil granulocytes in the lungs of RAG1KO mice. Although elevated neutrophil granulocyte numbers are typically associated with disease exacerbation, the majority of neutrophil granulocytes observed following SU5416 treatment remained within the lung vasculature, and their presence did not result in increased lung pathology. To assess whether neutrophil granulocyte accumulation after SU5416 treatment is specific to immunocompromised animals, we applied the same regimen to Mtb-infected immunocompetent C57BL/6 and B6.C3H-sst1 (“Kramnik”) mice. B6.C3H-sst1 is a congenic strain with the C3HeB/FeJ-derived susceptible allele at the sst1 locus on a C57BL/6J background, resulting in hypoxic, necrotic lesions similar to human lung granulomas [22,23]. We observed increased neutrophil granulocytes in the lung vasculature of both strains after treatment; however, we did not detect accelerated lung inflammation in either model.

Although further work is needed to completely elucidate the mechanism behind the biological effects of SU5416, this is the first preclinical/early translational study in immunocompromised mice to demonstrate that VEGFR1/2 blocking therapy reduces lung inflammation without affecting protection. Future studies will be needed to investigate if VEGFR1/2 blockade could be used as adjunct immunomodulatory therapy for immunocompromised human patients.

## 2. Materials and Methods

### 2.1. Animals

RAG1KO (RRID:IMSR_JAX:002216), wild-type C57BL/6J (RRID:IMSR_JAX:000664), Flt-1 (VEGFR1)-floxed (stock no: RRID:IMSR_JAX:028098) and LysM^Cre^ (RRID:IMSR_JAX:004781) mice were purchased from the Jackson laboratory (Bar Harbor, ME). B6J.C3-Sst1^C3HeB/FeJ^Krmn/Mmnc (shortly, B6.C3H-sst1 or “Kramnik”) mice (RRID:MMRRC_043908-UNC) were purchased from the MMRRC repository. VEGFR1^LysM-KO^ were generated by crossbreeding the VEGFR1-floxed and the LysM^Cre^ strains, and VEGFR1^LysM-KO^ mice were homozygous VEGFR1^flox/flox^ and carried one copy of the LysM^Cre^ alleles. VEGFR1^WT^ littermate controls were homozygous VEGFR1^flox/flox^ and did not carry the LysM^Cre^ allele. Animals were genotyped by Transnetyx (Cordova, TN, USA).

For all experiments, 8- to 28-week-old mice were used. Experimental units are (3–6 mice/group) age and sex-matched animals, divided either to treatment and control groups or to KO and WT littermate groups, as detailed in Supplementary Table S1. Mice were housed and bred in microisolator cages (maximum of 5 mice/cage) at the University of Wisconsin-Madison Medical Sciences Center animal facility (Madison, WI, USA).

### 2.2. Ethics Statement

The animal study protocol was approved by the Institutional Animal Care and Use Committee of University of Wisconsin Madison (protocol ID: M005644-R03, date of approval 5 September 2025). All experimental procedures were performed in accordance with the guidelines of the Institutional Animal Care and Use Committee. Exclusion criteria were as follows: animals that reached the humane end point (20% loss of body weight) during the experiment, or in which the IV or the IP injections were not performed successfully, were euthanized and excluded from the analyses, as established *a priori* to the experiments; data from all other animals is shown in the analyses. Experimental sample size was determined *a priori* using results from previous experiments [21] and based on NIH guidelines described by Charan and Kantharia [24]. We used the piface.jar application as suggested by the UW-Madison RARC “SOP for justifying the number of animals in research ACAPAC policy 2013-051”.

### 2.3. Infection with Mtb

*Mycobacterium tuberculosis* strain H37Rv (ATCC #27294), provided by Dr. Adel Talaat (University of Wisconsin Madison, Madison WI, USA), was electroporated with either pTEC27, a tdTomato-expressing plasmid (RRID:Addgene_30182) or with pTEC19, an E2-Crimson-expressing plasmid (RRID:Addgene_30178) and grown at 37 °C in Middlebrook 7H9 media (BD, Franklin Lakes, NJ, USA, Cat# 271310) supplemented with 10% OADC (Thermo Fisher Scientific, Waltham, MA, USA, Cat# B12351), 0.2% glycerol (Fisher Scientific, Hampton, NH, USA, Cat# BP229), 0.1% Tween 80 (MilliporeSigma, Burlington, MA, USA, Cat# P4780), and 100 μg/mL Hygromycin B Gold (InvivoGen, San Diego, CA, USA, Cat# ant-hg). For aerosol infections, Mtb H37Rv culture was taken during the mid-logarithmic phase, washed and diluted in PBS, and loaded into an Inhalation Exposure System (Glas-Col, Terre Haute, IN, USA, Model# 099C A4212). Mice were infected with a dose of aerosolized bacteria, previously titrated to result in approximately 200 colony-forming units (CFU) of bacilli in the lungs. Growth of Mtb, infections, and collection of tissue were done in a Biosafety Level 3 facility under approved biosafety guidelines. RAG1KO, C57BL/6J, VEGFR1^LysM-KO^ and VEGFR1^WT^ mice were infected for 4 weeks, and B6.C3H-sst1 mice were infected for 16 weeks due to the slower development of granulomatous pathology in this mouse strain.

### 2.4. Measurement of Bacterial Burden (CFU)

To determine the Mtb dose received at infection, 1–2 mice were harvested one day after inhalation exposure, and whole lungs were homogenized and plated to measure bacterial burden. At the time of harvest, the superior lobe of the right lung was used from each animal for CFU determination, as follows. The whole lungs were weighed, then the right superior lobe was dissected, weighed, and homogenized in 2 mL of phosphate-buffered saline (PBS, Corning, Corning, NY, USA, Cat# 21-040) using a GentleMACS Tissue Dissociator in M-tubes (Miltenyi Biotec, Gaithersburg, MD, USA, Cat# 130-093-236) and an Mtb-validated instrumental program. A logarithmic dilution series was then prepared from the sample and plated onto Middlebrook 7H10 agar medium (BD Biosciences, Franklin Lakes, NJ, USA, Cat# 262710) supplemented with 10% OADC (Thermo Fisher Scientific Cat# B12351) and 0.5% glycerol (Fisher Scientific Cat# BP229) and 50 μg/mL Hygromycin B Gold (InvivoGen Cat# ant-hg). Plates were incubated at 37 °C for 21 days, and the number of colonies on each plate was counted and reported as CFU per gram of lung tissue.

### 2.5. VEGF Receptor Inhibition

VEGF receptor was inhibited in vivo with SU5416 (Semaxanib, Cayman Chemical, Ann Arbor, MI, USA, Cat# 13342) by IP injections (25 mg/kg/day, from a 10 mg/mL stock solution in DMSO), starting at seven days prior to harvest. Control mice received equal volumes of vehicle (DMSO) IP. Daily administration of 25 mg/kg SU5416 has been shown to reduce granulomatous pathology [21] and inhibit tumor xenografts without significant toxicity in mice [25]. Stratified randomization was used to allocate animals into treatment groups: animals within same age range and sex were organized by weight and divided into groups in an alternating order to ensure similar weight distribution between the control and treatment groups. In order to minimize potential confounders, cage locations were assigned randomly and cages were swapped weekly. The order of tissue harvest from the two experimental groups was randomized. Since weight loss has been a reported side effect of SU5416, we monitored the animals and euthanized mice that lost 20% of their body weight during the treatment (humane end point).

### 2.6. In Vivo Intravascular Cell Staining

To discriminate between intravascular and tissue leukocytes, we used the intravascular CD45 labeling method because perfusion has been ineffective in removing all leukocyte populations, including neutrophils and granulocytes, from lung capillaries. Additionally, perfusion may alter lung architecture, which could reduce or eliminate non-vascular leukocyte populations. In contrast, CD45 intravascular staining effectively labels all vascular leukocytes, allowing us to study and differentiate between tissue and blood compartments within the same sample without the artifacts caused by perfusion [26,27]. In vivo intravascular cell staining was performed as previously described [27]. Shortly, mice were anesthetized with isoflurane and received a retro-orbital injection of 2.5 μg anti-mouse CD45.2 A647 (BioLegend, San Diego, CA, USA, Cat# 109818, RRID:AB_492870) or CD45.2 BV421 (BioLegend Cat# 109832, RRID:AB_2565511) antibody in 200 μL of sterile PBS three minutes before harvest. This effectively labelled ~99% of leukocytes in the blood.

### 2.7. Cell Isolation

Mice were euthanized with isoflurane overdose, and blood was collected into PBS + 20 U/mL heparin (Millipore Sigma, Burlington, MA, USA, Cat# H3149) and then centrifuged at 500× *g* for 12 min at 4 °C. RBCs were lysed with 10 mL NH_4_Cl solution (StemCell Technologies, Vancouver, BC, Canada, Cat# 07800) according to the manufacturer’s protocol. Cells were washed with 20 mL of HBSS (Gibco, Thermo Fisher Scientific, Waltham, MA, USA, Cat# 14170161) + 5% heat-inactivated (H.I.) FBS (Gibco Cat# A5256701) and counted in a 1 mL volume. For single-cell isolation from the superior and post-caval lobes of the right lungs, the lung lobes were chopped up with scissors then transferred into a gentleMACS C Tube (Miltenyi Biotec, Gaithersburg, MD, USA) containing 4 mL HBSS with calcium and magnesium (Gibco Cat# 24020117) + 50 U/mL DNase I (Sigma-Aldrich, St. Louis, MO, USA, Cat# D4513) + 0.1 mg/mL trypsin inhibitor (Sigma-Aldrich Cat# T6522). When all samples were collected, 2 mL of 2 mg/mL Type IV Collagenase (Millipore Sigma Cat# 11088866001) was added to the mixture, and the tissue was processed with a GentleMACS Dissociator (Miltenyi Biotec) using the Lung cell 1 program. After this, the samples were digested for 40 min in an orbital shaker incubator at 37 °C and 115 rpm and then processed with the GentleMACS Dissociator using the Lung cell 2 program. The cell suspension was pressed through a 40 µm mesh size cell strainer, washed with 10 mL HBSS + 5% H.I. FBS and then centrifuged at 300× *g* for 10 min at 4 °C. RBCs were lysed with 1 mL of NH_4_Cl solution according to the manufacturer’s protocol. Cells were washed with 10 mL of HBSS + 5% H.I. FBS and counted in a 1 mL volume.

### 2.8. Flow Cytometry

After cell isolation and generation of single-cell suspensions, 1 × 10^6^ cells were washed in PBS and incubated with Ghost Dye UV 450 (Tonbo Biosciences, San Diego, CA, USA, Cat# 13-0868) according to the manufacturer’s protocol. The cells were then washed with FACS buffer (PBS + 1% BSA + 0.05% sodium azide), then preincubated with Mouse BD Fc Block (BD Biosciences, Franklin Lakes, NJ, USA, Cat# 553142, RRID:AB_394657) according to the manufacturer’s instructions, and then stained with fluorophore-conjugated anti-mouse antibodies CD11b PerCP-Cy5.5 (Thermo Fisher Scientific Cat# 45-0112-82, RRID:AB_953558), Ly-6G Alexa 700 (BioLegend, San Diego, CA, USA, Cat# 127622, RRID:AB_10643269), CD49b PE (BD Biosciences Cat# 558759, RRID:AB_397108), and F4/80 FITC (Thermo Fisher Scientific Cat# 11-4801-81, RRID:AB_2735037) in 100 µL of FACS buffer for 30 min on ice, protected from light. Cells were washed with 1 mL of FACS buffer and were fixed with 1 mL of 4% PFA for 1h at 4 °C. Fixed cells were washed with FACS buffer, filtered through a 40 µm nylon mesh, measured with an LSRII flow cytometer (BD Biosciences) or an NL 3000 V/B/R spectral flow cytometer (Cytek Biosciences, Fremont, CA, USA) and analyzed with FlowJo v10 (BD Biosciences). Total cell numbers of immune cell populations in were calculated using the following formula: [(cell count in lung lobe used for flow cytometry/100) × percentage value of gated cell population]/(weight of lung lobe used for flow cytometry/weight of whole lung). Statistical analyses were performed using the GraphPad Prism v6 software.

### 2.9. Histological Quantifications (IHC, H&E)

The left lung lobe was fixed in 4% PFA at 4 °C for 24 h and then incubated in 30% sucrose at 4 °C overnight. Following this, the lungs were frozen in Tissue-Tek O.C.T. Compound (Sakura Finetek, Torrance, CA, USA) on dry ice. Sections measuring 20 µm were stained as follows: tissues were blocked and permeabilized with 1 mL of staining buffer (PBS with 1.0% BSA, 0.1–0.25% Triton X-100 and 0.05% NaN_3_) for 30 min at room temperature and then stained overnight at 4 °C with the primary antibodies biotin anti-mouse Ly-6G (BioLegend Cat# 127604, RRID:AB_1186108, 1:100 dilution), goat anti-mouse E-cadherin (R&D Systems, Minneapolis, MN, USA, Cat# AF748-SP, RRID:AB_355568, lot: CYG0423101, 1:200 dilution), rabbit anti-mouse VEGFA C-terminal [EP1176Y] (AbCam, Waltham, MA, USA, Cat# ab52917, RRID:AB_883427, lot: 35537-29, 1:200 dilution), goat-anti-mouse NKp46 (R&D Systems, Cat# AF2225-SP, RRID:AB_355192, lot: KWW0624091 in 1:75 dilution), rabbit anti-mouse VEGFR1 (Thermo Fisher Scientific, Cat# MA5-32045, RRID:AB_2809339, lot: XF3603533A in 1:200 dilution), and rat anti-mouse CD11b FITC (BD Biosciences, Cat#553310, RRID:AB_394774, lot: 6134771, in 1:100 dilution). After washing the sections three times for 5 min in staining buffer, sections were labelled for four hours at room temperature with secondary reagents Streptavidin, Alexa Fluor™ 555 (Thermo Fisher Scientific, Cat# S32355, 1:500 dilution), donkey anti-goat IgG A488 (Thermo Fisher Scientific Cat# 11055, RRID:AB_2534102, lot: 2747580, 1:500 dilution), donkey anti-rabbit IgG A647 (Thermo Fisher Scientific Cat# A31573, RRID:AB_2536183, lots: 1,874,788 and 2997084, 1:500 dilution), donkey anti-goat IgG A568 (Thermo Fisher Scientific Cat# A11057, RRID:AB_2534104, lot: 2776028, 1:500 dilution), and donkey anti-rat IgG A488 (Thermo Fisher Scientific Cat# A21208, RRID:AB_2535794, lot: 2892444, 1:500 dilution). The sections were washed three times for 5 min in staining buffer and one time for 5 min in PBS and then mounted in ProLong Gold Antifade Mountant (Thermo Fisher Scientific Cat# P36934) and imaged with a Spinning Disc Confocal Microscope equipped with Blue/Green/Red/Far Red filter sets for 4 color imaging (Nikon, Melville, NY, USA). IHC data was analyzed using the FIJI (ImageJ2, version 2.3.0/1.53f) and Imaris (version 9.8.2, Bitplane, Oxford Instruments, Concord, MA, USA) software. For neutrophil distribution, Ly-6G^+^ and IV CD45^+^ cells were identified, and then the Ly-6G^+^ IV CD45^+^ cell percentage was calculated among all Ly-6G^+^ cells.

For H&E analysis, the medial lobe of the right lung was fixed in 10% phosphate-buffered formalin for 24 h and then submitted to the UW-Madison Translational Research in Pathology (TRIP) Laboratory for processing and preparation of 5 μm thick, hematoxylin and eosin (H&E)-stained FFPE sections. Inflamed areas were identified by increased cellular infiltration and tissue consolidation, outlined and measured using the FIJI software, as a percentage of the total lung section area [21]. Each data point represents an animal, and the inflamed area index was calculated by dividing the value of each data point by the control group’s average. Statistical analyses were performed using the GraphPad Prism v6 software (GraphPad Software, Inc., San Diego, CA, USA). IHC and H&E analyses were performed on de-identified image files by lab members blinded to the treatments the animals received.

## 3. Results

### 3.1. VEGFR1/2 Blockade Attenuates Lung Inflammation and Monocyte Infiltration Without Affecting Bacterial Control in Immunocompromised Mice

Previous studies have demonstrated that VEGFR1/2 blockade reduces Mtb-induced lung pathology in immunocompetent mice [21]. Additionally, VEGFA functions as a potent chemokine that attracts monocytes, which subsequently differentiate into VEGFA-producing macrophages within lung granulomas. This process establishes a cycle that perpetuates excessive inflammation without conferring additional protection. The VEGFR1/2 inhibitor SU5416, but not selective VEGFR2 blockers, reduced tissue pathology in immunocompetent mice, suggesting a VEGFR1-dependent mechanism that regulates monocyte influx and reduces inflammation in the infected lungs without compromising protection, as measured by lung CFU burden [21].

**Figure 1:**
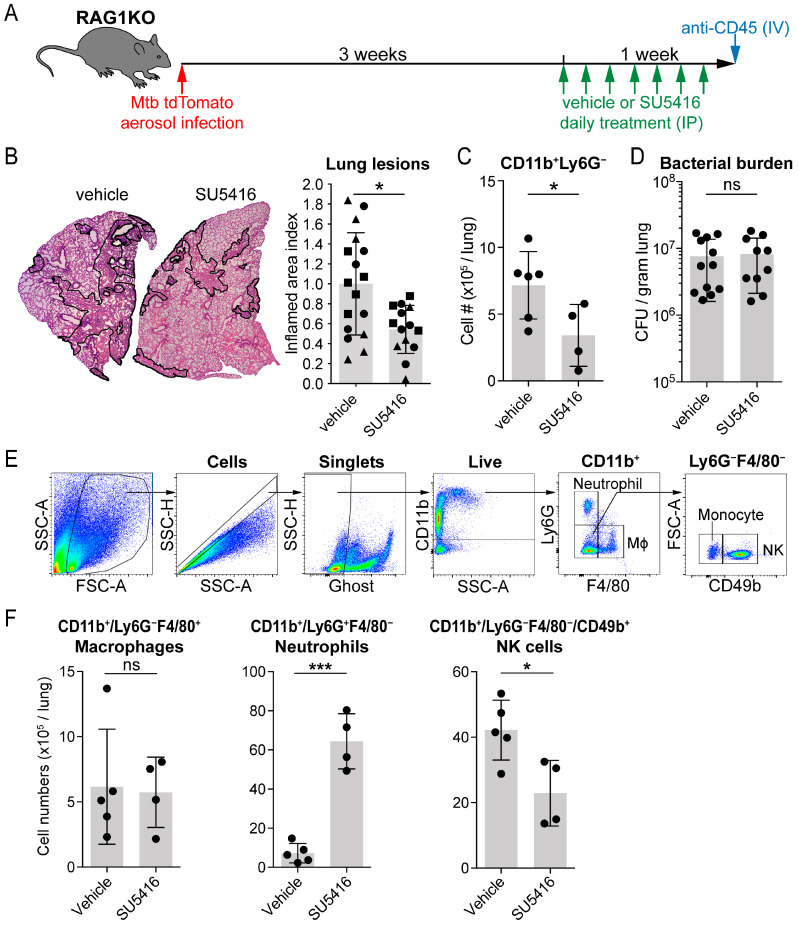
VEGFR1/2 blockade reduces lung inflammation and monocyte and NK cell infiltration without a change in the bacterial burden in immunocompromised (RAG1KO) mice. (**A**) Experimental outline. Over the course of three experiments, 33 mice RAG1KO mice of both sexes, between 8–22 weeks of age, were infected with ~200 CFU of aerosolized Mtb H37Rv for 4 weeks. During the last week, animals were treated with the VEGFR1/2 blocker SU5416 (25 mg/kg/day) or volume-matched vehicle (DMSO) IP. Ages and sexes were equally distributed between the control (N = 17) and treatment (N = 14) groups, as detailed in Supplementary Table S1. Two animals in the SU5416-treated group reached the predefined humane end point before the harvest and were therefore excluded from the analysis; all other animals were included in all analyses if the assay was performed in the experiment. (**B**) Lung inflammation was analyzed on H&E-stained sections of the middle lobe of the right lung by measuring the percentage of consolidated tissue areas on H&E-stained sections. To account for the inherent biological variability in the Mtb infection model, data were pooled from three experiments (data points from the same experiment are marked with similar markers: circles, squares or triangles), normalized to the control group’s median value and analyzed with the Mann–Whitney test (*p* = 0.0166); error bars represent mean ± SD. N = 17 vehicle-treated and N = 14 SU5416-treated mice are included. (**C**) Absolute numbers of CD11b+ Ly6G− cells in the lung tissue (in cell/singlet/live/IV CD45− cell gate). Representative data from one experiment is shown. Data was analyzed by an unpaired *t*-test assuming equal variances (*p* = 0.045); error bars represent mean ± SD. N = 6 vehicle-treated and N = 4 SU5416-treated mice are included; two mice were excluded from the SU5416-treated group as they reached the predefined humane end point before the harvest. (**D**) Bacterial burden was measured in the superior lobe of the right lung, homogenized and plated on 7H10 agar and counted after 3 weeks of growth at 37 °C. Data were pooled from two experiments and analyzed using the Mann–Whitney test (*p* = 0.8718); error bars represent mean ± SD. N = 12 vehicle-treated and N = 10 SU5416-treated mice are included; two mice were excluded from the SU5416-treated group as they reached the predefined humane end point before the harvest. (**E**) Gating strategy for macrophages (cell/singlet/live/CD11b+/Ly6G−F4/80+), neutrophil granulocytes (cell/singlet/live/CD11b+/Ly6G+F4/80−), and NK cells (cell/singlet/live/CD11b+/Ly6G−F4/80−/CD49b+). (**F**) Cell numbers of immune cell populations in the Mtb-infected lungs, calculated using the percentage of each cell population in the live gate, the lung weights and cell counts at lung harvest. Macrophages (*p* = 0.8703), neutrophils (*p* < 0.0001) and NK cells (*p* = 0.0195). Data was analyzed by an unpaired *t*-test, * 0.01 ≤ *p* < 0.05; *** *p* < 0.001; ns, not significant; error bars represent mean ± SD. Representative data from one experiment is shown, and data from all 9 experimental animals (N = 5 vehicle-treated and N = 4 SU5416-treated) are shown.

To test whether SU5416 treatment results in a similar beneficial effect in immunocompromised animals, we infected 8–22-week-old RAG1KO mice with 200 CFU of aerosolized Mtb H37Rv tdTomato-tagged bacteria for 4 weeks. During the last week of the infection, the animals received intraperitoneal (IP) injections of SU5416 (25 mg/kg/day) or volume-matched vehicle (DMSO) as a control (Figure 1A). We found significantly reduced lung pathology, as measured by the area of consolidated airways (Figure 1B), and fewer monocytes (CD11b + Ly6G− cells) in lung tissue (Figure 1C) in the SU5416-treated animals. The bacterial burden was similar between the VEGFR1/2-blocker-treated and the control groups, suggesting that the reduced monocyte influx does not affect bacterial protection in the immunocompromised hosts (Figure 1D).

After establishing that VEGFR1/2 blockade reduces monocyte recruitment in RAG1KO mice, the study next evaluated whether the treatment alters the abundance of other leukocyte populations in the Mtb-infected lungs of mice lacking an adaptive immune system. The gating strategy is specified in Figure 1E. The analysis shows that while CD11b + F4/80-Ly6G-expressing macrophage populations remained unaffected after VEGFR1/2 blockade, there was a significant decrease in the CD11b+ CD49b+ Ly6G− F4/80− NK cell population with the increase in CD11b+ Ly6G+ F4/80− neutrophil populations (Figure 1F).

Since it has been shown that perfusion is not an effective method for complete removal of immune cell populations from the lung capillaries [27], to elucidate if these cell populations reside in the lung tissue or in the lung vasculature, in the following experiments, we performed intravascular (IV) CD45 staining, administered 3 min before sacrificing the animals, to discriminate between blood and tissue leukocytes, instead of perfusing the lungs.

### 3.2. Following VEGFR1/2 Blockade, Neutrophil Granulocyte Recruitment to Mtb-Infected Lungs Increases in Immunocompromised Mice; However, Most Recruited Granulocytes Are Retained in the Lung Vasculature

In tuberculosis, neutrophils are early responders and mediators of late responses, inducing tissue damage. Our data showed that increased lung neutrophil accumulation did not worsen pathology or promote bacterial expansion in immunodeficient mice (Figure 1). To clarify why higher neutrophil levels had no effect on tissue pathology, we analyzed neutrophil distribution between lung tissue and vasculature.

**Figure 2:**
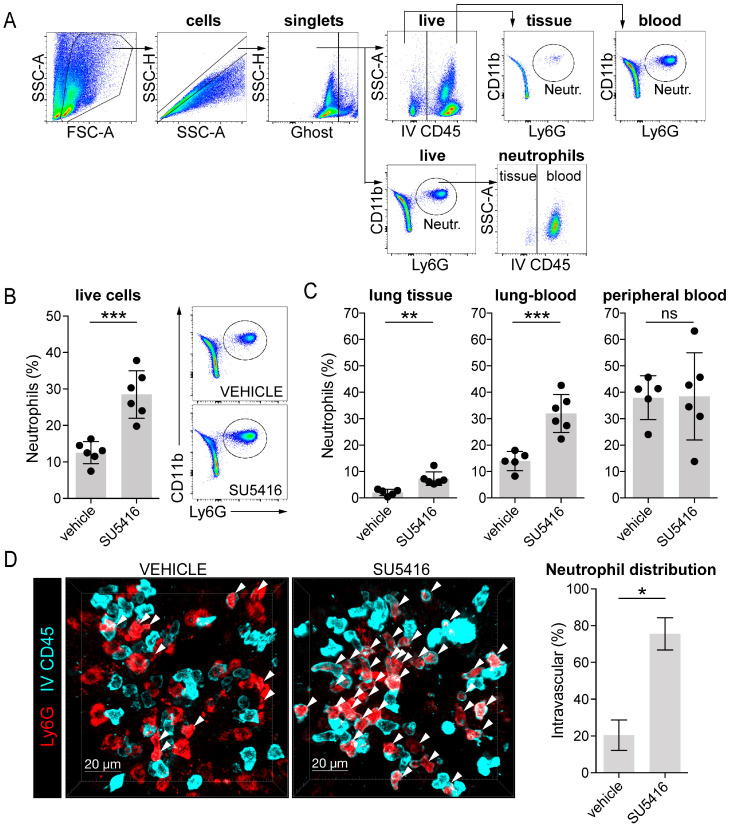
VEGFR1/2 blockade results in increased neutrophil granulocyte recruitment to Mtb-infected lungs in immunocompromised mice, but the vast majority of the recruited granulocytes remain in the lung vasculature. Twelve-week-old female RAG1KO mice were infected with ~200 CFU of aerosolized Mtb H37Rv for 4 weeks. During the last week, animals were treated with the VEGFR1/2 blocker SU5416 (25 mg/kg/day, N = 6 animals) or vehicle (DMSO, N = 6 animals). Three minutes before harvest, the mice received 2.5 μg of anti-CD45 antibody IV to label vascular leukocytes. All animals were included in the analyses except in Figure 2C, where one animal was excluded from the vehicle group due to being used as a negative control for IV CD45 staining. (**A**) Gating strategy for neutrophil granulocytes (CD11b + Ly6Ghi) in the lung tissue (IV CD45−) or lung blood (IV CD45+) compartments. (**B**) Percentage of neutrophil granulocytes are shown in the live cell gate in the Mtb-infected lungs (*p* = 0.0003). (**C**) Analysis showing the frequency of neutrophil granulocytes in the tissue (*p* = 0.0025), in the vascular compartments (*p* = 0.0007) in the lung, and in the peripheral blood (*p* = 0.9519). (**A**–**C**) Data is from one representative experiment of three and was analyzed by an unpaired *t*-test, * *p* < 0.05; ** *p* < 0.01; *** *p* < 0.001; ns, not significant; error bars represent means ± SD. (**D**) Lung sections were stained with Ly6G-biotin antibody and SA-Alexa555; white arrows mark neutrophil granulocytes in the vasculature (Ly6G+ IV CD45+). Neutrophil distribution was calculated as the percentage of vascular (IV CD45+) cells among neutrophils (Ly6G+). Two images per mice were analyzed using an unpaired *t*-test (*p* = 0.0231).

The gating strategy for flow-cytometric analysis of the homogenized lungs is shown in Figure 2A. Quantification of all neutrophils in the live cell gate (Figure 2B) shows an increase in neutrophil granulocytes in the SU5416-treated RAG1KO Mtb-infected lungs, similar to the previous experiment (Figure 1E,F). Using intravenous (IV) CD45 staining, we discriminated between neutrophils localized in the lung tissue (IV CD45−) and the population residing in the lung vasculature (IV CD45+). In both treatment groups, most neutrophil granulocytes were IV CD45+, indicating they were localized in the lung vasculature, not in the lung parenchyma. We found a slight increase in the percentage of neutrophils in the tissue in the SU5416-treated group; however, the neutrophil granulocyte ratio among other cell types and their increase in the SU5416-treated mice was much higher in the lung vasculature (Figure 2C). In contrast, there was no difference in the percentage of neutrophils in the peripheral blood (Figure 2C). IHC staining of lung tissues also confirmed that a larger percentage of neutrophil granulocytes remained within the lung vasculature (Ly6G+ IV CD45+) in the SU5416-treated group (Figure 2D). This data shows that the majority of recruited granulocytes remained in the lung vasculature following VEGFR1/2 blockade in the immunodeficient mice.

### 3.3. Monocyte-Produced VEGFA Is the Main Source of VEGFA in the Mtb Infected Lung

**Figure 3:**
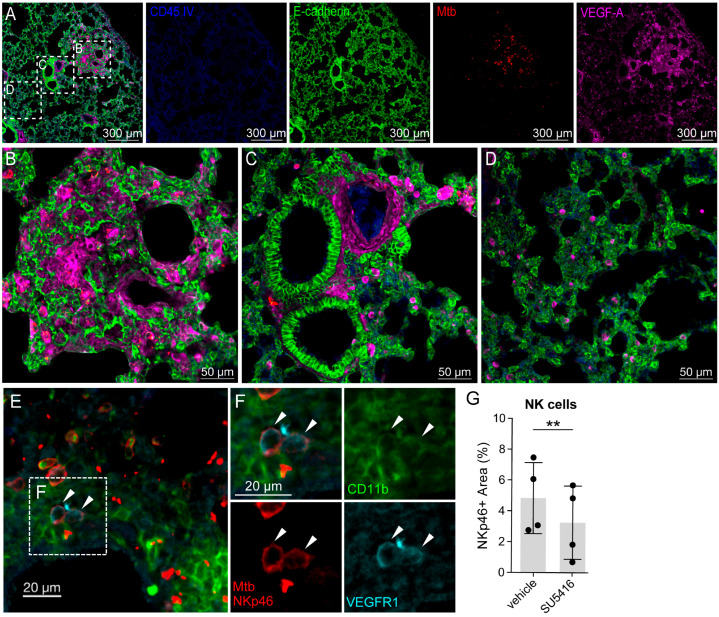
VEGF-A production in the Mtb-infected lungs is predominant in the lung lesions and endothelial and perivascular cells; a subset of NK cells expresses VEGFR1, and the number of NK cells decreases following SU5416-treatment in Mtb infected RAGKO mouse lung tissue. RAG1KO mice of both sexes that were 12–16 weeks old were infected with ~200 CFU of aerosolized Mtb H37Rv tdTomato for 4 weeks. During the last week, animals were treated with SU5416 (25 mg/kg/day) or vehicle (DMSO). Three minutes before harvest, the mice received 2.5 µg of anti-CD45 BV421 antibody IV to label vascular leukocytes. (**A**–**D**) Lung sections were stained with goat anti-mouse E-cadherin, rabbit anti-mouse VEGFA, donkey anti-goat IgG A488, and donkey anti-rabbit IgG A647. (**A**) Lung section is shown from a vehicle-treated mouse. VEGFA expression is highest in the Mtb-infected lesion areas (**B**) and perivascular areas (**C**). Compared to these regions, VEGFA production is significantly lower in E-cadherin+ airway epithelial cells in the uninflamed region (**D**) in the same lung section. (**E**–**G**) Lung sections were stained with goat-anti-mouse NKp46, rabbit anti-mouse VEGFR1, rat anti-mouse CD11b FITC donkey anti-goat-A568 (Invitrogen, Thermo Fisher Scientific, Waltham, MA, USA cat: A11057, lot: 2776028), donkey anti-rabbit IgG A647 and donkey anti-rat IgG A488. (**E**,**F**) CD11b+ cells (green) form aggregates in Mtb (red rods)-containing regions of RAGKO mouse lungs. NK cells (NKp46+, red) are among the CD11b+ cell population, and a subset of these cells express VEGFR1 (cyan). White arrowheads indicate IV CD45− CD11b+ VEGFR1+ NKp46+ cells in the lung parenchyma adjacent to Mtb-infected CD11b+ cells. Green: CD11b, red: Mtb tdTomato and NKp46, cyan: VEGFR1, blue: IV CD45 (no positive cells in the image). (**G**) The NKp46+ area is decreased in SU5416-treated mice. A total of 3–7 images per mouse from 4 vehicle- and 4 SU5416-treated, 13–17-week-old, sex-matched (3F, 1M/group) animals that were taken and analyzed using similar microscope settings pairwise from the two groups. Dots represent mean values of the analyzed image sets from each mouse. Data is analyzed by a paired *t*-test to compare treated and control animals that were imaged pairwise on the same day using the same instrument settings (*p* = 0.0046), ** 0.001 ≤ *p* <0.01; error bars represent mean ± SD. (F = female, M = male).

Since NK cell populations have been shown to express VEGFR1 [28,29], we hypothesized that granuloma-produced VEGFA might act as a chemoattractant for these cells as well and that VEGFR1 blockade might contribute to their decreased presence in the infected lungs. Although type II alveolar epithelial cells are important VEGFA producers in healthy lungs [30], we have demonstrated that granuloma macrophages are the most prominent source of VEGFA in Mtb-infected lungs in immunosufficient mice [21], and here, we show similar results in the Mtb-infected RAG1KO lungs (Figure 3A–D). This supports a similar, VEGFA-VEGFR1-mediated recruitment cycle in the immunocompromised animals. These data extend our previous results regarding the tissue-protective effect of VEGFR1 inhibition in immunosufficient mice to animals that lack an adaptive immune system and highlight the potential of VEGF blockade to reduce TB pathology in immunocompromised populations.

### 3.4. A Subset of NK Cells Express VEGFR1, and the Number of NK Cells Decreases Following SU5416 Treatment in Mtb-Infected RAGKO Mouse Lung Tissue

We also tested VEGFR1 expression in neutrophils that were able to migrate into the lung tissue. On lung sections from Mtb-infected RAG1KO mice that received intravascular CD45 staining before harvest, we identified VEGFR1-expressing NK cell populations within the lung tissue (CD11b+ NKp46+ VEGFR1+ IV CD45− cells) adjacent to infected Mtb tdTomato+ CD11b+ cell aggregates (Figure 3E,F), and we verified our flow cytometry results, showing a decreased NK cell presence in the lung tissue in SU5416-treated mice (Figure 3G).

### 3.5. VEGFR1 Expressed by Myeloid Cells Does Not Contribute to Neutrophil Granulocyte Recruitment to Mtb-Infected Lungs Following SU5416 Treatment

**Figure 4:**
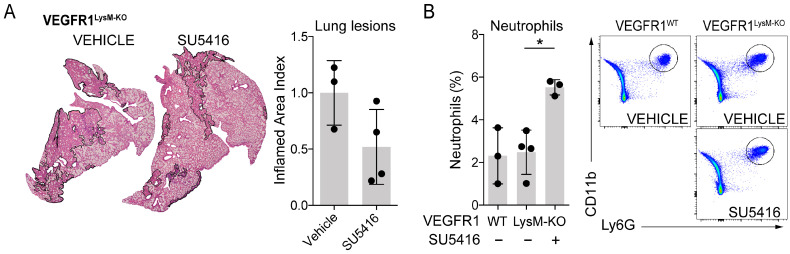
VEGFR1 expressed by myeloid cells does not contribute to neutrophil granulocyte recruitment to Mtb-infected lungs following SU5416 treatment. Flt1^flox/flox^ LysM^Cre/wt^ mice (VEGFR1^LysM-KO^) and Flt1^flox/flox^ LysM^wt/wt^ (littermate controls, VEGFR1^WT^) of both sexes that were 10–28 weeks old were infected with ~200 CFU of aerosolized Mtb H37Rv for 4 weeks. During the last week of infection, animals were divided into groups of 3–4 age- and sex-matched mice and treated with the VEGFR blocker SU5416 (25 mg/kg/day) IP or received vehicle (DMSO) IP. (**A**) Lung inflammation was analyzed on H&E-stained sections of the middle lobe of the right lung of vehicle- (N = 3, 1M, 2F, 27–28 wks old) or SU5416-treated (N = 4, 2M, 2F, 27–28 wks old) VEGFR1^LysM-KO^. Data was analyzed using an unpaired *t*-test (*p* = 0.1020); error bars represent mean ± SD. All experimental animals were included in the analysis. (**B**) The inferior and post-caval lobes of the right lungs were analyzed by flow cytometry from vehicle-treated VEGFR1^WT^ (N = 3, 2F, 1M, 10–13 wks old), vehicle-treated VEGFR1^LysM-KO^ (N = 4, 3F, 1M, 10–13 wks old) and SU5416-treated VEGFR1^LysM-KO^ (N = 4, 4F, 13–21 wks old) animals. One (13 wks old F) animal was excluded from the last group due to lung hemorrhage during the harvest, which led to outlier data in this animal. Percentages of neutrophil granulocytes are shown in the cell/singlet/live gate. Data was analyzed using ordinary one-way ANOVA with Tukey’s multiple comparisons test; * 0.01 ≤ *p* < 0.05; error bars represent mean ± SD. (F = female, M = male).

To clarify the role of myeloid VEGFR1 in neutrophil homing to inflamed Mtb-infected lungs, we infected VEGFR1^LysM-KO^ mice and their littermate controls (VEGFR1^WT^) with Mtb and then assessed lung inflammation and neutrophil accumulation following SU5416 treatment. SU5416 reduced lung pathology in VEGFR1^LysM-KO^ mice, as indicated by a lower inflamed area index compared to vehicle controls (Figure 4A). The percentage of neutrophil granulocytes was similar between vehicle-treated VEGFR1^LysM-KO^ mice and their littermate controls. However, SU5416 treatment resulted in a significant increase in lung neutrophil granulocytes in VEGFR1^LysM-KO^ mice compared to vehicle-treated controls of the same genotype (Figure 4B). These results indicate that the increased neutrophil presence in the lung vascular compartment after SU5416 treatment occurs through a mechanism independent of VEGFR1-mediated myeloid cell migration.

### 3.6. VEGFR1/2 Blockade Results in Increased Neutrophil Granulocyte Recruitment to Mtb-Infected Lungs in Immunocompetent Mtb-Resistant and Mtb-Hypersusceptible Mice

Given that VEGFR-blocker-treated immunocompromised mice exhibited reduced pathology and increased neutrophil granulocytes in Mtb-infected lungs, we quantified neutrophil granulocytes after SU5416 treatment in immunocompetent animals. We assessed neutrophil granulocyte numbers in the lungs of both C57BL/6J Mtb-resistant mice and B6.C3H-sst1 Mtb-hypersusceptible mice following SU5416 treatment. In the B6.C3H-sst1 strain, a 16-week infection period was used to allow for the development of hypoxic, necrotic granulomas resembling human lesions, a characteristic of Mtb-induced pathology in this model.

**Figure 5:**
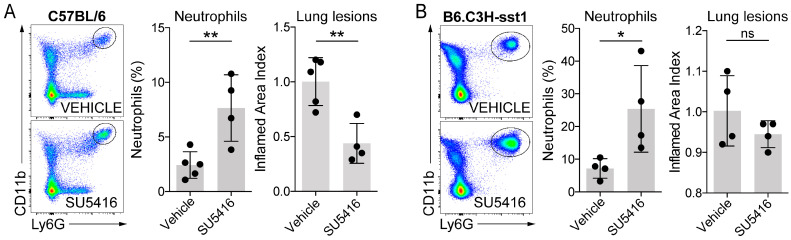
VEGFR1/2 blockade results in increased neutrophil granulocyte recruitment to Mtb-infected lungs in immunocompetent Mtb-resistant and immunocompetent Mtb-hypersusceptible mice. (**A**) Female 16-week-old C57BL/6 mice (N = 10) were infected with ~200 CFU of aerosolized Mtb H37Rv for 4 weeks. During the last week, animals were treated with SU5416 (25 mg/kg/day, N = 5) or received vehicle (DMSO, N = 5). Three minutes before harvest, the mice received 2.5 μg of anti-CD45 antibody IV to label vascular leukocytes. The inferior and post-caval lobes of the right lung were analyzed by flow cytometry; percentages of neutrophil granulocytes are shown in the live cell gate (unpaired *t*-test, *p* = 0.095). Lung inflammation was analyzed on H&E-stained sections of the middle lobe of the right lungs (unpaired *t*-test, *p* = 0.0045); ** 0.001 ≤ *p* < 0.01. One mouse was excluded from the analysis because it reached the *a priori* defined humane end point during the treatment. (**B**) B6.C3H-sst1 mice (N = 8) of both sexes that were 15 weeks old were infected with ~200 CFU of aerosolized Mtb H37Rv for 16 weeks. During the last week, animals were divided into sex-matched groups and treated with SU5416 (25 mg/kg/day, N = 4) or vehicle (DMSO, N = 4). Three minutes before harvest, the mice received 2.5 μg of anti-CD45 antibody IV to label vascular leukocytes. The inferior and post-caval lobes of the right lung were analyzed by flow cytometry; percentages of neutrophil granulocytes are shown in the live cell gate (unpaired *t*-test, *p* = 0.0363); * 0.01 ≤ *p* < 0.05; ns, not significant. Lung inflammation was calculated as a percentage of consolidated tissue areas on H&E-stained sections of the middle lobe of the right lungs normalized to the control group’s median (unpaired *t*-test, *p* = 0.2610). Data from all 8 animals is shown.

As in RAG1KO animals, SU5416 treatment increased neutrophil granulocyte percentages in both Mtb-resistant C57BL/6 mice (Figure 5A) and immunocompetent Mtb-hypersusceptible B6.C3H-sst1 (“Kramnik”) mice (Figure 5B). Baseline and post-treatment neutrophil levels were higher in B6.C3H-sst1 mice, reflecting the later infection stage and more severe inflammation in this strain (Figure 5B). Notably, although neutrophil accumulation increased in the blood and lungs of C57BL/6 mice, lung pathology significantly decreased (Figure 5A).

## 4. Discussion

Host-directed therapies meet the need for improved Mtb treatment by targeting the pathogen and reducing tissue damage caused by antibacterial immune responses [31,32,33,34,35,36,37,38]. We have previously shown that VEGFR blockade, or myeloid cell-specific knockout of the *vegfa* gene, reduced granulomatous pathology in Mtb-infected immunocompetent mice without compromising protection [21]. We got similar results following treatment with SU5416, a small-molecule receptor tyrosine kinase (RTK) inhibitor of VEGFR1 and VEGFR2 [21,39]. SU5416 has been shown to inhibit VEGFR-mediated tumor growth in mice [25] and has been tested in a phase III clinical trial against colorectal cancer [40], thus offering a promising adjunct therapy in TB-induced pathology.

We aimed to expand our previous data by assessing the effects of SU5416 treatment in Mtb-infected immunocompromised mice to evaluate its potential as an adjunctive therapy for TB in individuals with weakened adaptive immunity, such as those with TB-HIV coinfection. SU5416 treatment reduced Mtb-induced lung lesions and monocyte/macrophage infiltration in immunocompromised (RAG1KO) mice without affecting bacterial burden. These findings build on our previous results showing that VEGF-A secreted by granuloma macrophages drives excessive lung inflammation and that VEGFR1/2 blockade reduces TB pathology without impairing bacterial control.

An effective Th1 immune response and IFN-γ production is paramount in protection against tuberculosis. Individuals with compromised Th1 responses, such as HIV/AIDS patients, are susceptible to the development of active TB disease characterized by the loss of bacterial control and development of cavitary lung lesions. In RAG-deficient mice, lacking mature T and B cells, NK-cell-produced IFN-γ has been shown to provide temporary protection from Mtb infection and lung pathology [41]. Mtb-induced lesions are smaller in RAG-deficient animals compared to WT mice, but Mtb infection still leads to excessive lung inflammation. We used RAG1KO animals to study the effect of VEGFR1/2 blockade on Mtb-induced lung pathology and bacterial control to investigate if a similar treatment could serve to alleviate TB-induced pathology in immunocompromised patients. Since RAG1KO animals lack adaptive immune responses, the chronic stage of infection is not reached in this model as the animals succumb to the disease. Therefore, we aimed to study the effects of VEGFR1/2 blockade on the innate immune responses in this model at an early time point, 28 days post-infection. To complement our findings in the RAG1KO animals, we included experiments in immunocompetent C57BL/6 and B6.C3H-sst1 mice, the latter representing a chronic infection model (16-week infection), which partially addresses concerns about chronic disease representation.

We observed that both the absolute number and percentage of neutrophil granulocytes increased in the Mtb-infected lungs of RAG1KO animals after SU5416 treatment. Although neutrophils are typically associated with greater lung pathology, loss of bacterial control, and TB reactivation, we observed decreased lung pathology despite increased neutrophil accumulation. Using CD45 IV staining 3 min before euthanasia to distinguish intravascular from tissue leukocytes, we found that most neutrophils were located in the lung vasculature. To assess whether this effect was specific to immunocompromised animals, we treated Mtb-infected, immunocompetent C57BL/6 mice with SU5416 and observed a similar increase in lung neutrophil recruitment, accompanied by reduced lung pathology. We also repeated the infection and SU5416 treatment in Mtb-hypersusceptible B6.C3H-sst1 mice, which develop necrotic and hypoxic granulomas resembling human pathology [42,43]. In these mice, we detected a significant increase in neutrophil influx to the lung vasculature without increased lung inflammation. These findings indicate that SU5416-induced neutrophil homing to the lungs occurs independently of adaptive immune function and the type of granulomatous inflammation, and importantly, it does not compromise infection control or increase lung inflammation.

It has been previously shown that a subpopulation of human and mouse neutrophil granulocytes express VEGFR1. VEGF-A rapidly induces tissue recruitment of circulating neutrophils, and parallel activation of VEGFR1 on neutrophils and VEGFR2 on endothelial cells is required for circulating neutrophils to adhere to the endothelium and emigrate into the tissue. Interestingly, VEGF-A does not induce chemotaxis in either CD49d+ or CD49d− neutrophil subpopulations in mice [44,45]. These observations might explain the neutrophil granulocyte accumulation in the lung vasculature following SU5416 treatment in wild-type mice, since VEGF-A binding is not required for neutrophil chemotaxis toward the inflamed lungs, but engagement of both VEGFR1 on neutrophils and VEGFR2 on endothelial cells is required for neutrophil extravasation. However, our results in Mtb-infected vehicle-treated VEGFR1^LysM-KO^ and littermate control mice indicate that VEGFR1 expression was not required for the SU5416-induced neutrophil accumulation in the lungs. Our observation that the percentage of neutrophil granulocytes in the infected lung vasculature was increased following SU5416 treatment in VEGFR1^LysM-KO^ mice indicates that VEGFR1 does not drive neutrophil accumulation in lung vessels, and myeloid-cell-expressed VEGFR1 might not be needed in this process.

Our data show a significant decrease in CD11b+ CD49b+ Ly6G− F4/80− NK cell population following VEGFR1/2 blockade in Mtb-infected immunodeficient mice (Figure 1F). NK cells are important first responders with a direct cytolytic function and are an early and abundant source of IFN-γ in intracellular pathogen infections. IFN-γ activates macrophages, resulting in more effective pathogen killing, and causes maturation of dendritic cells, necessary for homing to the lymph nodes and antigen presentation to T cells to initiate the adaptive immune response [46]. An effective Th1 immune response and production of IFN-γ is important in protecting the host against tuberculosis [47]. The most important role of NK cells in Mtb infection is proposed to be through the early clearance of Mtb infection [48] through the recognition and lysis of Mtb and Mtb-infected cells [49,50]. At later stages of Mtb infection, when T cell responses are established, the role of NK cells might be redundant, as demonstrated by the fact that depletion of this cell population does not alter the bacterial load in the lungs of immunocompetent mice [51]. In immunocompromised RAG1KO mice lacking effective T cell responses, NK-cell-derived IFN-γ has shown to mediate important antimycobacterial effector functions and provide protection [41]. However, it has also been shown that Mtb and HIV infection each, as well as TB/HIV co-infection, can lead to the alteration in NK cell phenotype and function through the downregulation of activating NK cell receptors and expansion of functionally impaired NK cell subsets [52,53,54]. NK cells can also contribute to lung immunopathology, as increased NK cell-mediated cytotoxicity is associated with the presence of lung cavitation in pulmonary TB patients [55]. Reduced NK cell infiltration might contribute to the decreased lung inflammation in Mtb-infected RAG1KO mice following SU5416 treatment. VEGFR1 expression has been demonstrated in human NK cell lines [28]. Murine NK cells have been shown to express both membrane-bound and soluble VEGFR1, the latter functioning as a potent decoy receptor for VEGFA, inhibiting angiogenesis and VEGFA-mediated cell recruitment [29,56,57]. VEGFR blockade using monoclonal antibodies against VEGFR1 or VEGFR2 has yielded similar results to our data in a murine obliterative bronchiolitis model, namely, blunted NK cell activity reduced infiltration of CD11b+ cells (and, in their study, also CD4+ T cells) and downregulated mRNA expression of inflammatory cytokines, which resulted in attenuated development of obliterative airway disease [29]. Thus, VEGFR blockade might blunt NK cell responses directly by inhibiting their VEGFR1-mediated chemotaxis to the site of infection or indirectly via the reduction in monocyte infiltration and production of NK-cell-activating cytokines, such as IL-12, by tissue macrophages [29].

SU5416 (semaxanib) is a potent VEGFR2 inhibitor that blocks the VEGF-dependent kinase activity associated with the VEGFR2 (Flk-1/KDR) receptor, with a half-maximal inhibitory concentration (IC_50_) of 1.23 μM. The selectivity of SU5416 for VEGFR2 has been demonstrated by its 20-fold more potent inhibition of VEGFR than of PDGFRβ and its lack of activity against EGFR, insulin receptors and FGFR [39]. However, SU5416 has been shown to inhibit VEGFR1 in similar concentrations required to VEGFR2 blockade [58]. We have previously shown that, in contrast to SU5416, the VEGFR2-blocking antibody DC101 [59,60], while reducing the sinusoidal blood vessel density associated with infection, did not reduce the size and number of granulomas or number of granuloma leukocytes. Moreover, lesion size was also unaffected in Mtb-infected VEGFR2^LysM-KO^ mice that lacked VEGFR2 expression in their myeloid cells. In contrast, SU5416-treated or VEGFA^LysM-KO^ animals developed smaller lesions. Therefore, we hypothesized that SU5416 blockade reduces myeloid cell recruitment to the infected lungs through the VEGFR1 chemotactic receptor [21]. This hypothesis indeed explains the reduced percentage and number of monocytes and smaller inflamed areas in the SU5416-treated lungs. Interference with monocyte recruitment could result in altered neutrophil recruitment and licensing to enter the tissue, as it has been shown that Ly6C+ and Ly6C− monocyte populations contribute to the regulated recruitment of neutrophil granulocytes in urinary tract infection [61].

This study is limited by the absence of mechanistic experiments explaining how VEGFR blockade alters immune cell recruitment and why bacterial burden remains unchanged despite altered cellular infiltration, warranting further investigation. In previous studies, we extensively analyzed the cytokine profiles of granuloma-associated immune cells. We demonstrated that in immunocompetent mice, despite decreased inflammation, the cellular composition and proportion of cell types within the granuloma remained remarkably similar to control groups following anti-VEGFR1/2 treatment. The activation of T cells, measured by LFA-1 expression and production of interferon (IFN)-γ, was also unchanged despite VEGF-A inhibition. However, SU5416-treated mice had altered expression of several key cytokines, including CCL2, CCL4, CCL7, CCL12, CCL22, CXCL6, XCL1, interleukin (IL)-1β, IL-2, IL-6, and IL-10, tumor necrosis factor alpha (TNF-α) and IL-1α, but these changes were not significant. Based on this data, the changing cytokine/chemokine profile opened more questions rather than explaining the improved lung pathology [21]. The role of VEGFA–VEGFR1 (FLT1) signaling in monocyte chemoattraction is well-established in the literature [62,63,64,65], providing a mechanistic framework for the observed reduction in monocyte infiltration following SU5416 treatment. The unchanged bacterial burden despite altered immune cell composition likely indicates partial redundancy among innate immune effector mechanisms that control Mtb, consistent with previous findings [66,67,68,69,70]. Although this study achieved its goal to test if VEGFR1/2 blockade limits Mtb-induced pathology in immunocompromised mice, future studies incorporating multiplex cytokine/chemokine profiling (e.g., IL-6, TNF-α, IL-1β, CXCL chemokines) from lung homogenates and functional characterization of the NK cell population (i.e., TNF-α, IFN-γ, IL-4 and IL-10 cytokine production) would be essential to characterize the inflammatory mediator landscape following VEGFR blockade and to define the mechanisms underlying preserved bacterial control despite altered immune cell composition. In vitro VEGFR blockade assays and adoptive transfer experiments would be valuable to mechanistically validate these findings. The pattern recognition receptor, Receptor for Advanced Glycation End-products (RAGE), plays an important part in inflammatory signaling, and its role in tuberculosis-associated lung pathology is an emerging area of interest. Since RAGE signaling sustains inflammation, it has been linked to increased neutrophil infiltration and enhanced lung tissue injury [71,72]. RAGE is highly expressed on alveolar epithelial cells, macrophages, endothelial cells, and neutrophils, marking it an important target for both protein-level assessment (e.g., immunohistochemistry, ELISA) and mRNA expression profiling (e.g., qRT-PCR) in lung tissue from SU5416-treated and control Mtb-infected mice in future studies to assess the impact of SU5416 treatment on RAGE-mediated effects.

Although animals were age and sex matched for individual experiments, this study used a wide age range of animals and combined sexes and used a low sample size for certain experiments. Given the known sex-based differences in granuloma formation and immune responses in TB [73,74], to aid in the interpretation of the data, Supplementary Table S1 shows all data points used for the analyses, indicating the sex and age of corresponding animals. Although the results show the most robust impacts of SU5416 treatment, future studies with adequately powered, age- and sex-stratified cohorts are warranted to assess age- and sex-based differences and reduce variability.

The SU5416 treatment window used in this study represents a relatively short intervention period in the context of TB disease progression, and it therefore may not capture the long-term effects of VEGFR blockade on lung pathology, fibrosis, or granuloma stability. This was a deliberate design choice to assess the acute effects of VEGFR1/2 blockade on innate immune cell recruitment and early lung pathology and minimize potential confounding effects of prolonged VEGFR blockade on vascular homeostasis and wound healing. The short survival time of RAGKO animals (~45–50 days) following low-dose aerosol infection with the Mtb H37Rv strain [75,76] was also a limitation that did not allow for long-term treatments. Future studies employing extended treatment regimens at different stages of infection in immunocompetent animals (early vs. established disease) would be valuable to determine the optimal therapeutic window. This study was designed as a proof-of-concept investigation of VEGFR blockade as a host-directed therapy (HDT), independent of antibiotic treatment, to isolate the immunomodulatory effects of SU5416 from direct antibacterial effects. We acknowledge that in a clinical context, VEGFR blockers would be used as an adjunctive therapy alongside standard anti-tuberculosis antibiotics (e.g., isoniazid, rifampicin). The interaction between SU5416 and antibiotic therapy on immune responses and lung pathology remains to be investigated as an important next step toward clinical translation.

The primary focus of this study was on pulmonary disease, and systematic evaluation of extra-pulmonary organs (e.g., spleen, liver, kidneys, lymph nodes) for Mtb dissemination was not performed. However, extra-pulmonary dissemination of Mtb is particularly relevant in the context of (1) immunocompromised RAG1KO mice, which lack adaptive immunity, potentially facilitating hematogenous spread of Mtb beyond the lungs, and (2) because VEGFR blockade with SU5416 could theoretically influence vascular permeability and thereby alter the pattern of Mtb dissemination. We acknowledge the absence of extra-pulmonary assessment as a limitation of this study and recommend that future studies include bacterial cultures of spleen, liver, and lymph nodes to quantify extra-pulmonary bacterial burden, histological examination of extra-pulmonary organs for evidence of granuloma formation or tissue damage, and assessment of whether SU5416 treatment influences the pattern or extent of Mtb dissemination in immunocompromised hosts. These analyses would provide a more complete picture of the safety and efficacy of VEGFR blockade in the context of systemic Mtb infection.

To assess lung pathology, the lesion area was measured from H&E staining of a single lung lobe (consistently the same lobe across all animals), which allowed for a comparison of lung pathology between the treatment groups and allowed for gaining multiplex data (histology, flow cytometry, CFU) from the same animals. This approach is consistent with standard methodologies used in murine TB lung pathology studies [77,78,79]. However, data from one lung lobe may not comprehensively reflect the total pulmonary disease burden. Therefore, future studies that incorporate whole-lung sectioning or micro-CT imaging, such as that performed by Ordonez et al. [80], would provide a more comprehensive assessment of pulmonary disease burden.

In summary, our results show that VEGFR1/2-blocking therapy effectively reduces excess lung inflammation by inhibiting VEGFR1-mediated recruitment of monocytes and NK cells, without compromising host protection in Mtb-infected immunodeficient mice. While SU5416-treated animals showed increased neutrophil granulocytes in the lungs, most remained within the lung vasculature, likely due to inhibited VEGFR2-mediated extravasation. These findings support further investigation of VEGFR blockade as a potential adjunctive therapy to anti-tubercular regimens for immunocompromised populations, including Mtb/HIV patients.

## 5. Conclusions

This is a study demonstrating that VEGFR1/2 blockade reduces lung inflammation in immunocompromised TB models. The data suggest that SU5416 could be explored as an adjunctive immunomodulatory therapy alongside anti-tuberculosis drugs for immunocompromised patients. Further studies are needed to fully elucidate the mechanism behind the biological effects of SU5416 and investigate applicability in immunocompromised human patients. The data suggests that VEGFR blockade with SU5416 shows promise as a host-directed therapy for TB, particularly in immunocompromised individuals, by reducing harmful lung pathology while preserving protective immunity.

## Data Availability

The original contributions presented in this study are included in the article/Supplementary Material. Further inquiries can be directed to the corresponding author.

## Supplementary Materials

The following supporting information can be downloaded at: https://www.mdpi.com/article/10.3390/cells15070573/s1, Table S1: Experimental data points by mouse sex and age at infection.

## Abbreviations

H.I.	Heat-inactivated
IC_50_	Half-maximal inhibitory concentration
Mtb	*Mycobacterium tuberculosis*
PLCγ	Phospholipase Cγ
RTK	Receptor tyrosine kinase
TB	Tuberculosis
VEGFR	Vascular endothelial growth factor receptor
VEGF	Vascular endothelial growth factor
