# Supplementary material for: VEGFR Blockade Reduces Mycobacterium tuberculosis-Induced Lung Pathology in Immunocompromised Mice
Source: Cells. 2026 Mar 24;15(7):573. doi: 10.3390/cells15070573 (PMC13072297; doi:10.3390/cells15070573)
Supplement: Supplementary file 1 [file cells-15-00573-s001.zip › cells-4167520-supplementary.pdf]

Table S1. Experimental data points by mouse sex and age at infection

Lung Lesions (data from Figure 1B)

treatment	N	sex	age (wk)	value	treatment	N	sex	age (wk)	value
Vehicle	1	F	12.4	0.452	SU5416	1	F	12.4	0.708
	2	F	12.6	1.197		2	F	12.4	0.363
	3	F	12.6	1.779		3	F	12.7	0.195
	4	F	12.7	0.700		4	F	12.6	0.545
	5	F	13.0	0.545		5	F	13.0	0.586
	6	F	13.0	1.328		6	F	12.4	0.799
	7	F	10.3	1.071		7	F	10.3	0.531
	8	F	10.3	0.894		8	F	10.3	0.606
	9	F	10.3	0.698		9	F	10.3	0.782
	10	F	10.3	1.013		10	F	10.3	0.878
	11	F	10.3	1.326		11	F	22.4	0.043
	12	F	22.4	0.243		12	F	22.4	0.769
	13	F	22.4	0.496		13	M	22.6	0.440
	14	M	22.4	1.453		14	M	22.6	0.376
	15	M	22.4	1.840					
	16	M	22.4	1.650					
	17	M	22.4	0.318					

CD11b+Ly6G- cell numbers / lung (data from Figure 1C)

treatment	N	sex	age (wk)	value	treatment	N	sex	age (wk)	value
Vehicle	1	F	22.4	4.75E+05	SU5416	1	F	22.4	7.73E+04
	2	F	22.4	3.71E+05		2	F	22.4	4.88E+05
	3	M	22.4	7.99E+05		3	M	22.6	2.23E+05
	4	M	22.4	7.83E+05		4	M	22.6	5.78E+05
	5	M	22.4	1.07E+06					
	6	M	22.4	8.07E+05					

Bacterial burden (data from Figure 1D)

treatment	N	sex	age (wk)	value	treatment	N	sex	age (wk)	value
Vehicle	1	F	22.4	1.64E+07	SU5416	1	F	22.4	3.53E+06
	2	F	22.4	1.69E+07		2	F	22.4	9.27E+06
	3	M	22.4	1.31E+07		3	M	22.6	1.59E+07
	4	M	22.4	1.47E+07		4	M	22.6	1.82E+07
	5	M	22.4	5.45E+06		5	F	12.4	4.73E+06
	6	M	22.4	8.67E+06		6	F	12.4	1.93E+06
	7	F	12.4	1.68E+06		7	F	12.7	1.39E+07
	8	F	12.6	5.62E+06		8	F	12.6	9.22E+06
	9	F	12.6	2.60E+06		9	F	13.0	1.61E+06
	10	F	12.7	2.46E+06		10	F	12.4	3.58E+06
	11	F	13.0	2.17E+06					
	12	F	13.0	2.00E+06					

Macrophages - cell numbers (x10^5/lung) (data from Figure 1F)

treatment	N	sex	age (wk)	value	treatment	N	sex	age (wk)	value
Vehicle	1	F	10.3	13.69	SU5416	1	F	10.3	7.54
	2	F	10.3	5.82		2	F	10.3	2.17
	3	F	10.3	3.88		3	F	10.3	8.06
	4	F	10.3	2.32		4	F	10.3	5.17
	5	F	10.3	5.12					

Neutrophils - cell numbers (x10^5/lung) (data from Figure 1F)

treatment	N	sex	age (wk)	value	treatment	N	sex	age (wk)	value
Vehicle	1	F	10.3	14.83	SU5416	1	F	10.3	71.63
	2	F	10.3	6.35		2	F	10.3	56.36
	3	F	10.3	8.96		3	F	10.3	80.35
	4	F	10.3	2.30		4	F	10.3	49.38
	5	F	10.3	3.74					

NK cells - cell numbers (x10^5/lung) (data from Figure 1F)

treatment	N	sex	age (wk)	value	treatment	N	sex	age (wk)	value
Vehicle	1	F	10.3	53.31	SU5416	1	F	10.3	32.52
	2	F	10.3	47.45		2	F	10.3	13.61
	3	F	10.3	28.84		3	F	10.3	30.53
	4	F	10.3	41.50		4	F	10.3	14.95
	5	F	10.3	39.82					

Neutrophils - % in live cells (data from Figure 2B)

treatment	N	sex	age (wk)	value	treatment	N	sex	age (wk)	value
Vehicle	1	F	12.4	12.50	SU5416	1	F	12.4	19.80
	2	F	12.6	14.50		2	F	12.4	33.10
	3	F	12.6	11.50		3	F	12.7	37.80
	4	F	12.7	13.10		4	F	12.6	26.00
	5	F	13.0	7.48		5	F	13.0	24.00
	6	F	13.0	16.30		6	F	12.4	30.30

Neutrophils - % in lung tissue (data from Figure 2C)

treatment	N	sex	age (wk)	value	treatment	N	sex	age (wk)	value
Vehicle	1	F	12.4	3.28	SU5416	1	F	12.4	6.11
	2	F	12.6	2.56		2	F	12.4	12.30
	3	F	12.7	2.79		3	F	12.7	5.25
	4	F	13.0	0.38		4	F	12.6	6.76
	5	F	13.0	1.42		5	F	13.0	7.18
						6	F	12.4	5.97

Neutrophils - % in lung-blood (data from Figure 2C)

treatment	N	sex	age (wk)	value	treatment	N	sex	age (wk)	value
Vehicle	1	F	12.4	13.60	SU5416	1	F	12.4	22.30
	2	F	12.6	16.00		2	F	12.4	36.50
	3	F	12.7	13.90		3	F	12.7	42.60
	4	F	13.0	8.24		4	F	12.6	29.10
	5	F	13.0	18.00		5	F	13.0	27.40
						6	F	12.4	34.00

Neutrophils - % in peripheral blood (data from Figure 2C)

treatment	N	sex	age (wk)	value	treatment	N	sex	age (wk)	value
Vehicle	1	F	12.4	37.50	SU5416	1	F	12.4	13.80
	2	F	12.6	24.00		2	F	12.4	34.50
	3	F	12.7	41.40		3	F	12.7	42.70
	4	F	13.0	41.20		4	F	12.6	30.90
	5	F	13.0	45.70		5	F	13.0	45.70
						6	F	12.4	63.20

Neutrophils - % in lung tissue (data from Figure 2D)

treatment	N	sex	age (wk)	value	treatment	N	sex	age (wk)	value
Vehicle	1	M	22.4	14.629	SU5416	1	M	22.6	69.321
	2	M	22.4	26.301		2	M	22.6	81.698

NK cells - % NKp46+ area (data from Figure 3G)

treatment	N	sex	age (wk)	value	treatment	N	sex	age (wk)	value
Vehicle	1	F	13.0	3.065	SU5416	1	F	13.0	1.821
	2	F	13.0	2.745		2	F	13.0	0.690
	3	F	13.0	6.061		3	F	16.6	4.831
	4	M	13.0	7.464		4	M	16.6	5.674

Lung Lesions (data from Figure 4A)

treatment	N	sex	age (wk)	value	treatment	N	sex	age (wk)	value
Vehicle	1	M	27.1	1.099	SU5416	1	M	27.1	0.280
	2	F	28.0	0.677		2	M	27.1	0.218
	3	F	27.1	1.224		3	F	28.0	0.927
						4	F	28.0	0.651

Neutrophils (%) (data from Figure 4B)

group	N	sex	age (wk)	value	group	N	sex	age (wk)	value	group	N	sex	age (wk)	value
VEGFR1 WT + Vehicle	1	F	9.6	1.00	VEGFR1 LysM-KO + Vehicle	1	F	9.6	2.65	VEGFR1 LysM-KO + SU5416	1	F	20.6	5.80
	2	F	13.0	3.63		2	F	9.6	3.49		2	F	20.6	5.65
	3	M	13.0	2.30		3	F	9.6	1.02		3	F	13.0	5.12
						4	M	13.0	2.74					

Neutrophils (%) (data from Figure 5A)

treatment	N	sex	age (wk)	value	treatment	N	sex	age (wk)	value
Vehicle	1	F	15.9	1.07	SU5416	1	F	15.9	6.72
	2	F	15.9	2.49		2	F	15.9	10.78
	3	F	15.9	1.65		3	F	15.9	9.25
	4	F	15.9	4.29		4	F	15.9	3.83
	5	F	15.9	2.64					

Lung Lesions (data from Figure 5A)

treatment	N	sex	age (wk)	value	treatment	N	sex	age (wk)	value
Vehicle	1	F	15.9	1.090	SU5416	1	F	15.9	0.408
	2	F	15.9	0.716		2	F	15.9	0.288
	3	F	15.9	1.196		3	F	15.9	0.700
	4	F	15.9	0.815		4	F	15.9	0.345
	5	F	15.9	1.183					

Neutrophils (%) (data from Figure 5B)

treatment	N	sex	age (wk)	value	treatment	N	sex	age (wk)	value
Vehicle	1	M	15.4	7.09	SU5416	1	M	15.4	13.50
	2	M	15.4	10.50		2	M	15.4	27.70
	3	F	15.4	7.83		3	F	15.4	17.30
	4	F	15.4	3.29		4	F	15.4	43.10

Lung Lesions (data from Figure 5B)

treatment	N	sex	age (wk)	value	treatment	N	sex	age (wk)	value
Vehicle	1	M	15.4	1.099	SU5416	1	M	15.4	0.975
	2	M	15.4	0.915		2	M	15.4	0.899
	3	F	15.4	1.051		3	F	15.4	0.944
	4	F	15.4	0.935		4	F	15.4	0.975
